# Porcine Deltacoronavirus-Related Viruses in House Sparrows

**DOI:** 10.3390/v17101326

**Published:** 2025-09-30

**Authors:** Daoqun Li, Jiayu Xu, Elizabeth M. Ames, Mingde Liu, Bikash Aryal, Maria Chellis, Ramon Zegpi Lagos, Christopher M. Tonra, Qiuhong Wang

**Affiliations:** 1 Center for Food Animal Health, Department of Animal Sciences, College of Food, Agricultural and Environmental Sciences, The Ohio State University, Wooster, OH 44691, USA; 2 Department of Pathogen Biology, School of Clinical and Basic Medical Sciences, Shandong First Medical University & Shandong Academy of Medical Sciences, Jinan 250117, China; 3 Department of Veterinary Preventive Medicine, College of Veterinary Medicine, The Ohio State University, Columbus, OH 43210, USA; 4 School of Environment and Natural Resources, The Ohio State University, Columbus, OH 43210, USA

**Keywords:** porcine deltacoronavirus, sparrow deltacoronavirus, coronavirus, gammacoronavirus

## Abstract

Porcine deltacoronavirus (PDCoV) is an emerging enteric pathogen in pigs and a newly recognized zoonotic coronavirus in humans. Genetic analyses suggest that PDCoV originated from avian deltacoronaviruses, with sparrow deltacoronaviruses (SpDCoVs) being the most closely related. House sparrows (*Passer domesticus*) frequently visit farms and interact directly with pigs in barns, raising the possibility of interspecies transmission. We hypothesized that PDCoV can be transmitted between pigs and house sparrows. To investigate this, 200 house sparrows near Ohio swine farms were sampled and screened for gammacoronaviruses and deltacoronaviruses using RT-PCR targeting the conserved RNA polymerase region. Deltacoronaviruses and gammacoronaviruses were detected in 18.0% (36/200) and 5.5% (11/200) of fecal samples, respectively. Genomic sequence analysis of representative samples revealed that SpDCoVs are closely related to, but not direct ancestors of, PDCoVs. These SpDCoVs appear to be widespread in the U.S. Midwest and may contribute to PDCoV evolution. Attempts to isolate SpDCoV from these samples in embryonated chicken eggs and four cell lines were unsuccessful. Because coronaviruses frequently cross species barriers to cause epidemics and/or pandemics in humans and livestock, these findings underscore the need for ongoing surveillance of deltacoronaviruses in diverse wild animals, livestock, and humans to safeguard public health.

## 1. Introduction

Porcine deltacoronavirus (PDCoV) belongs to the *Deltacoronavirus* genus within the *Orthocoronavirinae* subfamily of *Coronaviridae* family (https://ictv.global/taxonomy. Last accessed on 17 July 2025). The viral genome is approximately 25.4 kb and encodes 14–16 nonstructural proteins (nsps), four structural proteins [spike (S), envelope (E), membrane (M), and nucleocapsid (N) proteins] and accessory proteins NS6, NS7 and NS7a [1,2]. It was first reported in 2012 that PDCoV was detected from the fecal samples collected from clinically normal pigs in Asia, but its etiological role was not identified until 2014 when it caused gastroenteritis outbreaks in pigs in the United States (U.S.) [2,3,4]. To date, it has been detected in the U.S., Canada, and many Asian countries [1]. Coronaviruses (CoVs) are positive-sense RNA viruses and infect various mammalian and avian species, causing enteric, respiratory, or systemic diseases of variable severity [5]. The *Coronaviridae* family contains four genera: *Alphacoronavirus*, *Betacoronavirus*, *Gammacoronavirus* and *Deltacoronavirus*. Recently, a new CoV was detected in the European badger and was proposed to form a new genus *Epsilon* [6]. These viruses evolve through an accumulation of point mutations and recombination. The later may allow a virus to create new forms that can infect different species [7]. AlphaCoVs and betaCoVs infect mammalian species, and gammaCoVs and deltaCoVs mainly infect birds. GammaCoVs were also detected from some whales [8]. All known deltaCoVs, except PDCoV, exclusively infect birds. Besides pigs, PDCoV has been detected in Asian leopard cats and Chinese ferret badgers [9]. It also experimentally infected calves, turkey and chicken poults, ducks, geese, ferrets and mice [10,11,12,13,14]. Most importantly, it was also detected from three Haitian children with self-limited illness (fever, respiratory symptoms, and/or abdominal pain), making it a zoonotic pathogen [15]. Molecular clock analysis suggested that PDCoV emerged in the 1990s [16] and global PDCoVs share high genetic identity (>96%) at the genomic level [1], suggesting its newly emerging status. Sequence analyses suggest that PDCoV probably evolved from multiple recombination events among avian deltaCoVs, such as sparrow deltaCoV (SpDCoV) HKU17, pigeon deltaCoV [17], munia CoV HKU13, bulbul CoV HKU11, and quail CoV HKU30 strains [1,18].

House sparrows (*Passer domesticus*) are opportunistic feeders and are closely associated with swine farms due to the availability of food and nesting sites. Whether PDCoV can be transmitted between sparrows and pigs is unknown. However, such information is important to understand the ecology and evolution of PDCoVs. In this study, we captured and sampled house sparrows (*Passer domesticus*) near Ohio swine farms and identified PDCoV-related CoVs from the fecal samples. Our results suggest that house sparrows contribute to the complexity of PDCoV evolution.

## 2. Materials and Methods

### 2.1. Sample Collection, Screening, and Preparation

House sparrows were trapped, captured, and sampled following the approved IACUC protocol. After sample collection, the birds were released. During the winter of 2020–2021, we collected the fecal/cloacal swab and oropharyngeal swab samples from 198 clinically healthy house sparrows captured in mist nets or baited cage traps near eight Ohio swine farms, which tested positive for PDCoVs by the Ohio Department of Agriculture (ODA) (Table 1). Two additional house sparrow samples were collected in the Clintonville neighborhood of Columbus, Ohio. Samples were diluted tenfold with virus transport medium (VTM) [HBSS (Invitrogen, Carlsbad, CA, USA) supplemented with 2% of fetal bovine serum (FBS, HyClone, Logan, UT, USA) and 1% of Anti-Anti (Invitrogen)], vortexed, and then centrifuged at 2000× *g* at 4 °C for 30 min. Subsequently, aliquots of the supernatants were made and stored at −80 °C until further analysis.

### 2.2. Virus Isolation in Embryonated Chicken Eggs (ECEs) and Four Cell Lines

We used 4- to 10-day-old specific pathogen-free (SPF) ECEs from The Ohio State University flock for virus isolation. The sparrow fecal suspension was filtered through 0.22 μm-pore size filters (Millipore, Chicago, IL, USA). The SF80 fecal suspension was also pretreated with a 1:100 dilution of the hyperimmune serum raised against turkey CoV (TCoV) (a gift from Dr. Mo Saif, The Ohio State University) overnight at 4 °C. For each ECE, 100–200 μL of the sparrow fecal sample was inoculated into the allantoic sac or chorioallantoic membrane (CAM), and ≥4 ECEs per sample per inoculation route, as previously reported [19,20] or illustrated by Jove video [21]. Phosphate-buffered saline without Mg^2+^ and Ca^2+^ [PBS(−)] (Gibco, Carlsbad, CA, USA) was used as a negative control and PDCoV FD22 strain (GenBank accession no. MZ291567.1; 7.8 log_10_ TCID_50_/mL) at 1:100 dilution was the positive control [22]. Then, the ECEs were incubated at 37 °C and 55–60% relative humidity for 5–7 days post-inoculation (dpi) or when the ECE was found dead. After putting the ECEs at 4 °C for 2–6 h, the allantoic fluids were harvested and centrifuged at 2000× *g* for 30 min at 4 °C to remove the cells and debris.

African green monkey kidney cell line Vero E6 (ATCC No. CRL-1586) was cultured in Dulbecco’s modified Eagle’s medium (DMEM) in the presence of 10% of FBS (Hyclone, Logan, UT, USA) and PSG (100 U/mL penicillin, 100 μg/mL streptomycin, and 2 mM of L-glutamine). The porcine kidney epithelial cell line LLC-PK1 (ATCC No. CL-101), swine testicle (ST) fibroblast-like cell line (ATCC No. CRL1746), and chicken embryo fibroblast cell line DF-1 (ATCC No. CRL-12203) cells were cultured in minimum essential medium (MEM) plus 5% FBS and the PSG, advanced MEM plus 5% FBS and PSG, and DMEM plus 10% FBS and the PSG, respectively [19,22]. For virus isolation, Vero E6, LLC-PK1, ST, and DF-1 cells were seeded in 48-well plates at densities of ~100,000 cells/well. Upon reaching > 95% confluence, 50 μL of allantoic fluid was mixed with 50 μL of serum-free medium supplemented with trypsin (final concentration 5 μg/mL) was added into each well. After 1 h adsorption at 37 °C with gently shaking every 15 min, 300 μL of maintenance medium containing 5 μg/mL of trypsin was added. The plate was incubated for four days. Cultures were examined daily for cytopathic effects (CPE), and the plate was frozen and thawed one time before harvesting both cells and supernatants. The RNA was extracted from the cell lysates using TRIzol reagent and viral RNA was tested by PDCoV RT-qPCR. All, except FBS, reagents were from Thermo Fisher Scientific (Waltham, MA, USA).

### 2.3. Concentration of CoVs from the Allantoic Fluids

The allantoic fluids (~38 or 13 mL), which tested as weak positives for PDCoV RNA by RT-qPCR (see below), were combined and clarified by centrifugation at 2000× *g* for 30 min, followed by filtration of the supernatants through 0.45 µm-pore size filters. Then, these samples were ultracentrifuged at 106,750× *g* for 2 h at 4 °C with or without 20% (*w*/*v*) sucrose cushion using an ultracentrifuge (Beckman Coulter, Miami, FL, USA). The pellet was resuspended in 200 µL of MEM for RNA extraction.

### 2.4. Viral RNA Extraction

Viral RNA was extracted from 50 μL of the field sparrow fecal or oropharyngeal sample suspension, the allantoic fluids and cell culture suspension using the 5× MagMAX-96 Viral Kit (Invitrogen, Carlsbad, CA, USA) and the MagMax™ Express machine (Applied Biosystems, Bedford, MA, USA), following the manufacturer’s instructions. Finally, 50 μL of RNA was obtained in an elution buffer. TRIzol (Invitrogen, Carlsbad, CA, USA) was also used to extract RNA from the concentrated allantoic fluid samples by ultracentrifugation as mentioned in Section 2.3.

### 2.5. Conventional Reverse Transcription (RT)-PCR and TaqMan Real-Time RT-PCR (RT-qPCR)

Pan-deltaCoV forward primer (5′-TGGGATTAYCCYAAGTGTGA-3′) and reverse primer (5′-CADGCDACACCRTCATCWG-3′, located in nucleotide position 13217–13236 and 13659–13641 of deltaCoV HKU30 strain (GenBank accession no. LC364345), and pan-gammaCoV forward primer (5′-TATGAWGGYGGYTGTATMCC-3′) and reverse primer (5′-GCYCTATCACACTTWGGATARTC-3′, located in nucleotide position 13806–13825 and 14230–14208 of gammaCoV TCoV-ATCC strain (GenBank accession no. EU022526), were designed based on the conserved RNA-dependent RNA polymerase (RdRp) region of avian deltaCoVs and gammaCoVs after performing sequence alignment (see below). The Qiagen OneStep RT-PCR Kit (Qiagen, Valencia, CA, USA) was used. The reaction mixture (25 μL) consisted of 5 μL of sample RNA or water (negative control) or positive control RNA (PDCoV RNA for deltaCoVs and TCoV RNA for gammaCoVs), 10 units of RNAsin (Promega), 5 μL of 5× PCR buffer, 1 μL of deoxynucleotide triphosphates (dNTPs) (10 mM each), 1 μL of enzyme mix, 1 μL each of forward and reverse primers (100 µM each), and RNase-free water. The RT was performed at 50 °C for 30 min followed by inactivation at 95 °C for 15 min and then PCR reaction with 40 cycles (94 °C for 30 s, 50 °C for 30 s, and 72 °C for 30 s), followed by a final extension step at 72 °C for 10 min. The RT-PCR products (443 bp for deltaCoVs and 425 bp for gammaCoVs) were analyzed using 1.5% agarose gel electrophoresis to confirm the proper sizes of amplicons.

The PDCoV RT-qPCR was performed as previously reported using Qiagen OneStep RT-PCR kit [3], targeting the conserved M gene of PDCoVs with forward primer 5′-ATCGACCACATGGCTCCAA-3′, reverse primer 5′-CAGCTCTTGCCCATGTAGCTT-3′, and probe 6-carboxyfluorescein-CACACCAGTCGTTAAGCATGGCAAGCT-IABkFQ. All primers and probe were synthesized by Integrated DNA Technologies (https://www.idtdna.com/page/about; Last accessed on 17 July 2025).

### 2.6. Sequencing

To sequence the panDelta and panGamma RT-PCR products of representative samples, which showed strong bands by agarose electrophoresis, the RT-PCR products of the correct size were purified using the QIAquick Gel extraction kit (Qiagen, Valencia, CA, USA) for direct Sanger sequencing at The Ohio State University Medical Center. To obtain the nearly complete genomes of SpDCoV SF3, SF80 and SF184, viral RNA was reverse transcribed to cDNA using the SuperScript™ IV cDNA synthesis kit (Invitrogen, Carlsbad, CA, USA) employing a strategy combining oligo(dT) and random hexamers’ priming. Subsequently, viral sequences were amplified using conventional PCR with the primer-walking method. The cDNA was amplified with the primers targeting the DeltaCoVs’ conserved regions and virus-specific primers designed based on the obtained viral sequences. PCR was performed using the high-fidelity PrimeSTAR GXL DNA polymerase (TaKaRa, Toshima-ku, Tokyo, Japan). To obtain additional sequences of SF80, nested PCR was also performed using PDCoV-specific primers targeting other than the 362-nt RdRp region. The purified PCR products were sent for Sanger sequencing. Finally, the overlapping DNA sequences were assembled for the near complete genomes.

### 2.7. Sequence Analysis

The Sanger sequence data was visualized using Chromas (version 2.6.6, https://technelysium.com.au/wp/chromas/; Last accessed on 17 July 2025). Reads generated from both forward and reverse primers were assembled and primer sequences were removed using the Lasergene software package (v10) (DNASTAR Inc., Madison, WI, USA). The Basic Local Alignment Search Tool (BLAST) was used to identify homologous matches at NCBI (version 2.17.0, https://blast.ncbi.nlm.nih.gov/Blast.cgi; Last accessed on 17 July 2025). Sequence alignment was performed using ClustalW, and maximum likelihood phylogenetic trees were constructed using Tamura-Nei model and with 1000 bootstrap replicates using MEGA 12.0 [23].

### 2.8. Prediction of S Protein 3D Structure

For comparative structural analysis, the amino acid sequences of the S proteins of SpDCoV/SF3, SpDCoV/SF184, and PDCoV/FD22 (GenBank accession numbers XUM38528, XXQ18308, and QWX20050, respectively) were retrieved. Structure prediction was performed using AlphaFold2 (ColabFold version 1.5.5, https://colab.research.google.com/github/sokrypton/ColabFold/blob/main/AlphaFold2.ipynb; Last accessed on 17 July 2025) [24]. Each prediction employed the PDCoV S protein structure (PDB ID: 9DEZ) as a template using an unpaired-paired alignment strategy with six recycles to optimize model quality. For each sequence, five top-ranked models were generated. The top three models were optimized using the Amber force field to reduce steric clashes and improve overall geometry. Finally, the best model was chosen for monomer illustration and for overlay of SpDCoV and PDCoV S proteins. The AlphaFold outputs included per-residue confidence (pLDDT) scores and predicted TM (pTM) scores, which evaluate the accuracy of SF3, SF184 and FD22 S structure against that of PDCoV (PDB ID: 9DEZ). Using the FD22 S protein as a reference, SF3 and SF184 S proteins were individually superimposed onto FD22, respectively, to obtain the overlays using the MatchMaker in ChimeraX (version 1.10.1) [25]. Two types of overall Root Mean Square Deviation (RMSD) values (all-atom RMSD and pruned-atom RMSD) were calculated to assess the similarity between the predicted S protein structures of SF3 and SF184 and that of FD22 structure, respectively. The pruned-atom RMSD was calculated based on a subset of atoms used for structural alignment, excluding less similar or flexible regions. RMSD quantifies the overall spatial deviation between two structures, and lower values indicate higher structural similarity. All structures were visualized with ChimeraX. Similarly, porcine aminopeptidase N (pAPN, GenBank No. QIS62296) was modeled using AlphaFold2 with the crystal structure of pAPN (PDB: 4FKE) as template, and the modeled pAPN together with the S proteins of SF3 and SF184 were subjected to multibody docking using HADDOCK v2.4 (version 2.4, https://rascar.science.uu.nl/haddock2.4/; Last accessed on 17 July 2025) [26]. We selected the corresponding active binding residues of S proteins based on the PDCoV S protein, which were the residues 318F, 320E, 322R, 357R, 396W, 397N, and 398Y [27]. We also defined the active residues (311Y, 374K, 421E, 424W, 739E, 742E and 786H) of pAPN. All passive residues were set to default. The SpDCoV SF3 and SF184 contain some of these residues. Then, the PRODIGY (protein binding energy prediction) webserver (https://rascar.science.uu.nl/prodigy/; Last accessed on 17 July 2025) was used to calculate the binding affinity, expressed in dissociation constant (Kd), at 38.6 °C, which is the physiological internal body temperature of pigs, between the S and the APN receptor.

### 2.9. Nucleotide Sequence Accession Numbers

The GenBank accession no. for the SpDCoVs identified in this study are listed: PV691851 (SF3) and PV935252 (SF184). The GenBank accession no. for the viruses included in the phylogenetic analyses are listed in the phylogenetic trees.

### 2.10. Statistical Analysis

Chi-square test was used to compare the prevalence of gammaCoVs and deltaCoVs in fecal samples.

## 3. Results

### 3.1. Detection of deltaCoVs and gammaCoVs from House Sparrows

We detected 36 deltaCoV-positive (18.0%, 36/200) and 11 gammaCoV-positive (5.5%, 11/200) samples, among which four samples (SF17, SF54, SF60, and SF80) were both positive for deltaCoV and gammaCoV, from the 200 house sparrows’ fecal/cloacal swab samples by the one-step RT-PCR assays (Table 1). The prevalence of deltaCoVs was significantly higher than that of gammaCoVs (*p* < 0.001, χ^2^ = 60.125 and 1 degrees of freedom). The 95% confidence interval (CI) for the prevalence (18%) of deltaCoVs is 12.7–23.3%, which is much higher than the prevalence (5.5%) of gammaCoVs. The first 100 house sparrow oropharyngeal swab samples were negative. Therefore, we did not test the rest of 100 oropharyngeal swab samples. Two of the 11 gammaCoV-positive and 18 of the 36 deltaCoV-positive samples’ RT-PCR products were sequenced and BLAST confirmed their identity. Both the gammaCoVs SF60G (329-nt) and SF80G (298-nt) shared 99% nucleotide identity with the gammaCoV TCoV-ATCC strain and were not further characterized in this study. Among the deltaCoVs, 17 SpDCoVs were highly similar, sharing 98–100% nucleotide identity, and genetically closely related to quail deltaCoV HKU30 strain (93–95% identity) in the partial RdRp region (349–362-nt). Interestingly, SpDCoV strain SF80 shared 100% nucleotide identity with many PDCoV strains, including the FD22 strain in our laboratory, in this partial RdRp region (362 nt). To rule out potential contamination with the positive control PDCoV, we tested again the original sample, confirming that sample SF80 contained both a TCoV-like gammaCoV and a PDCoV-like deltaCoV. The PDCoV-like virus consistently produced only a faint band on agarose gel following RT-PCR, indicating an extremely low level of deltaCoV RNA in the sparrow droppings and suggesting inefficient replication in the sparrow.

### 3.2. Genomic Analysis of SpDCoV Strains SF3 and SF184

**Figure 1:**
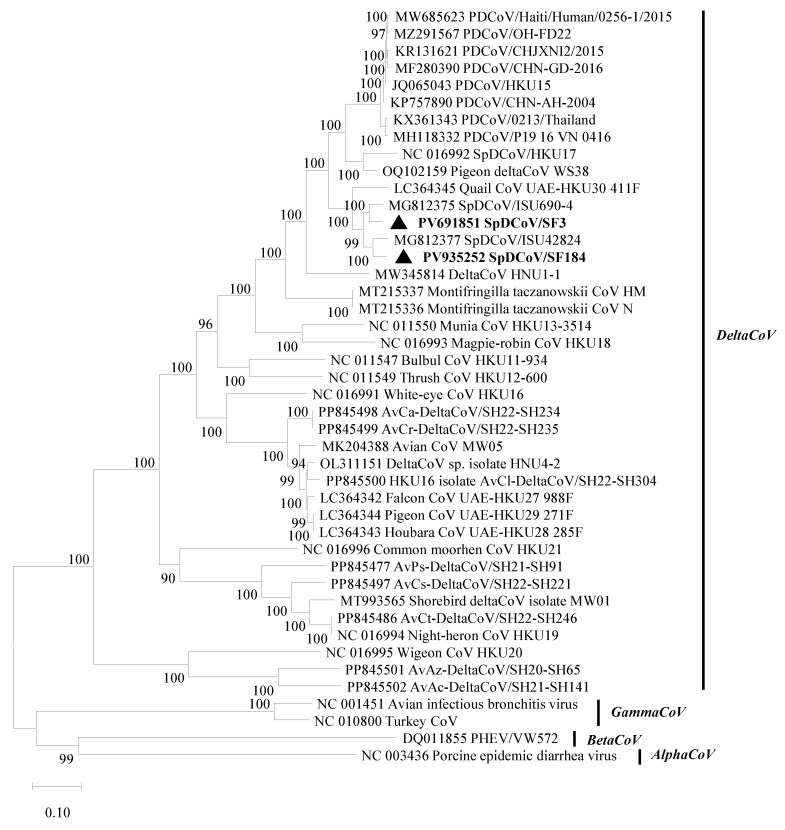
Phylogenetic tree of SpDCoV genomes using the maximum likelihood method with 1000 bootstrap replicates. The SpDCoVs detected in this study are highlighted in bold and marked with a solid black triangle. Several alphaCoVs, betaCoVs and gammaCoVs were included as outgroup controls. The GenBank accession no. of each virus is listed at the beginning of each strain.

**Figure 2:**
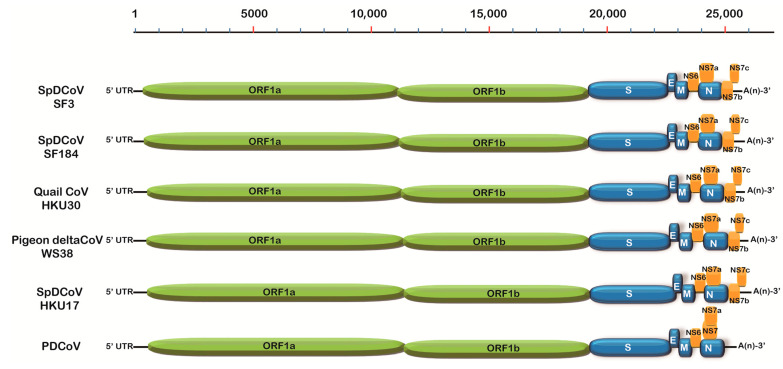
The genomic organization of PDCoV (GenBank: KT336560), SpDCoV/SF3 (GenBank: PV691851), SpDCoV/SF184 (GenBank: PV935252), avian deltaCoVs: quail CoV HKU30 (GenBank: LC364345), pigeon deltaCoV WS38 (GenBank: OQ102159), and SpDCoV/HKU17 (GenBank: NC 016992). The nucleotide scale is located on the top.

The near-complete genomes of two SpDCoVs (SF3 and SF184) were obtained. We could not obtain additional sequences beyond the partial RdRp region for SF80 although ultracentrifugation of the PDCoV RNA-positive allantoic fluids was attempted to concentrate potential virus particles and RT followed by nested PCR was performed to amplify additional genomic regions. Phylogenetic analyses showed that SF3 and SF184 grouped with the US SpDCoV strains (ISU690-4, ISU690-7, ISU73347 and ISU42824), which were detected from house sparrows at swine barns/farms in Illinois or Minnesota in 2017 [28] (Figure 1). These SpDCoVs were genetically closest to quail deltaCoV HKU30. Comparison of genomic organization also showed that SpDCoVs possess four accessory proteins—NS6, NS7a, NS7b, and NS7c—similar to other avian deltaCoVs, whereas PDCoV contains NS6 along with co-terminal NS7 and NS7a (Figure 2). In ORF1ab, E, M, and N trees, these SpDCoVs clustered together and were closely related to PDCoV and HKU17 strains (Supplementary Figure S1A–D).

### 3.3. The S Proteins of SpDCoVs Have Specific INDELs Compared with PDCoVs

**Figure 3:**
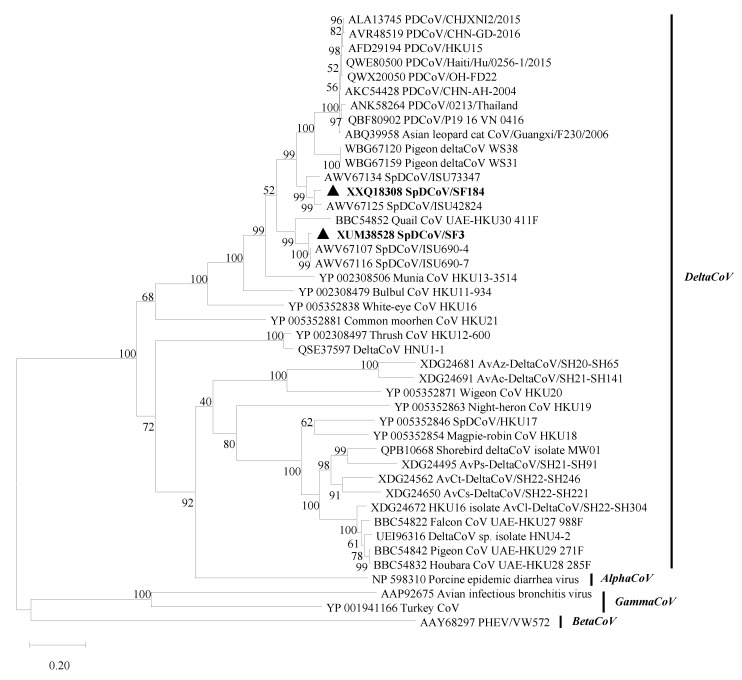
Phylogenetic analysis of the spike proteins of CoVs using the maximum likelihood method with 1000 bootstrap replicates. The SpDCoVs detected in this study are bolded and indicated with black triangles. The GenBank accession no. of each virus is listed at the beginning of each strain.

**Figure 4:**
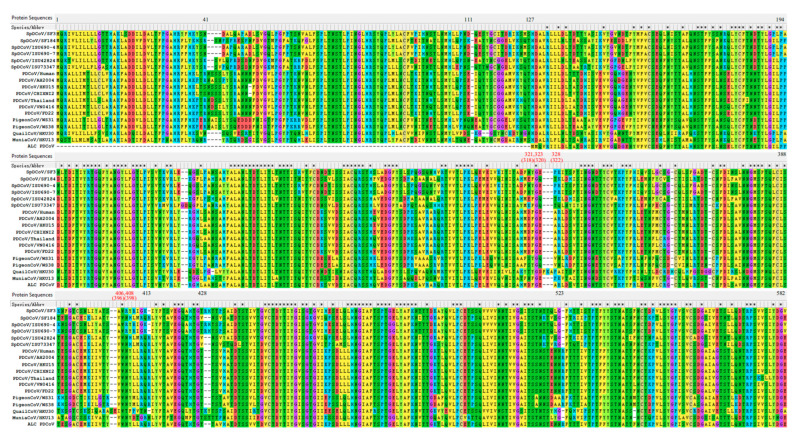
Sequence alignment of the S protein S1 subunit of PDCoVs, SpDCoVs, pigeon deltaCoVs, quail deltaCoV HKU30, munia deltaCoV HKU13, and Asian leopard cat (ALC) PDCoV. The location of PDCoV critical binding residues to pAPN are marked (red), with the corresponding location in PDCoV S1 in parentheses. The * indicate all viruses share the same residue at the position.

In the S protein, SpDCoV HKU17 only shares 46% identity with PDCoVs. The phylogenetic tree of the S proteins (Figure 3) showed that pigeon deltaCoVs WS38 and WS31 are genetically closest to PDCoVs, sharing 82.7–83.7% amino acid identity (Supplementary Table S1) [17]. Among known SpDCoVs strains, SF184, ISU42834 and ISU73347 SpDCoVs share the highest sequence identity (81.5–82.6%) with the earliest detected PDCoV strain AH2004 (GenBank: KP757890), followed by SF3, ISU690-4 and ISU690-7 SpDCoVs (75.0–75.5%). Quail HKU30 (72.5%), munia HKU13 (71.6%), and bulbul HKU11 (70.3%) also share high sequence identity with PDCoVs in the S protein. Compared with point mutations, insertion-deletions (INDELs) are less frequent. Sequence alignment of the S1 subunit of PDCoVs and genetically related avian deltaCoVs revealed PDCoV-specific INDELs in the S1 region (Figure 4), including the insertions of 41NS42 and 523R, which may reflect viral adaptation from avian to mammalian hosts. Insertions of 43SN44 and 413R and a 2-amino-acid-Del of 428RH429 in SF184-like SpDCoVs suggest that these viruses may be the potential intermediate forms in the interspecies transmission of PDCoV from sparrows to pigs. Interestingly, the recently identified pigeon deltaCoV WS38 and WS31 contain all the PDCoV-specific INDELs in the S1 subunit. However, WS38 and WS31 have a specific one amino acid insertion of 111N. The corresponding nucleotide sequence alignments of S1 region is provided in Supplemental Table S3.

PDCoV uses pAPN as the main host receptor but may also utilize other receptors, as pAPN-knockout ST cells still support limited PDCoV replication [29]. The C-terminal domain (CTD) of the S1 subunit of PDCoV S protein, spanning amino acid residues 300–419, contains the receptor-binding domain (RBD) that interacts with the conserved domain II and domain IV of pAPN [27,30]. Ji et al. identified five critical residues 318F, 320E, 322R, 396W and 398Y of PDCoV, corresponding to residues 321F, 323E, 328R, 406W and 408Y in the aligned sequences, for the receptor binding, with 396W and 398Y the most essential [27] (Figure 4). Using AlphaFold2, high-quality 3D structures of the pAPN were generated with pLDDT scores of 93.8 and pTM scores of 0.899. To investigate the interaction between S proteins of SpDCoVs and pAPN, molecular docking studies were performed using HADDOCK 2.4. The best clusters were chosen based on the lowest RMSD and most negative Z and HADDOCK scores. The best models were listed in Supplementary Table S4. Interface residues involved in interactions from the S of SpDCoVs and the pAPN are listed Supplementary Table S5. The predicted SF3 S and pAPN binding Kd was 12 nM, which is higher than that (2.6 nM) between SF184 and pAPN, suggesting that SF184 has a higher binding affinity to pAPN than SF3.

### 3.4. Prediction of S Protein 3D Structures Revealed That SpDCoV SF184 Strain Is Closer to PDCoV than SF3

**Figure 5:**
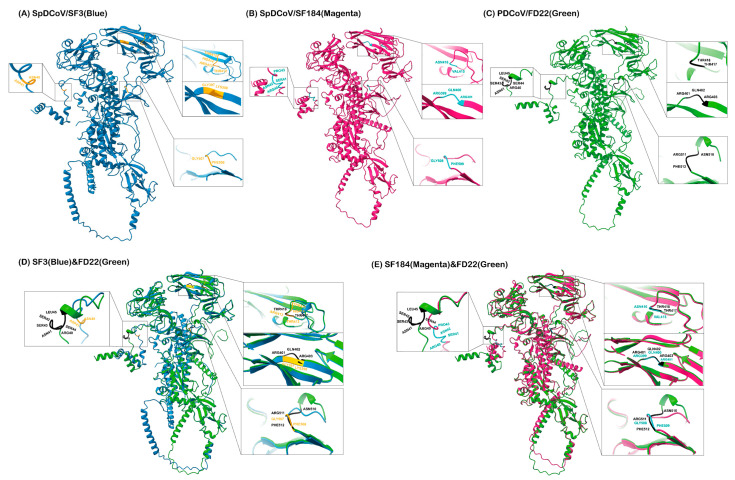
The predicted 3D structure of the S monomer of SpDCoV SF3 (**A**), SF184 (**B**), and PDCoV FD22 (**C**), and the overlay of SF3 and FD22 (**D**) and SF184 and FD22 (**E**). Specific amino acid residues 40–46, 411–413, 426–429, and 521–523 in the aligned sequences in Figure 4 correspond to SF3 residues 40–41, 397–398, 412–415, and 507–508 (labeled in the amplified squares of (**A**,**D**)), SF184 residues 40–43, 399–401, 415–416, and 508–509 (labeled in the amplified squares of (**B**,**E**)), and FD22 residues 40–45, 401–403, 417–418, and 510–512 (labeled in the amplified squares of (**C**)).

Using AlphaFold2, high-quality 3D structures of the S proteins of SpDCoVs SF3 (pLDDT scores of 82.0; pTM scores of 0.663) and SF184 (pLDDT scores of 82.5; pTM scores of 0.672) strains and PDCoV FD22 strain (pLDDT scores of 84.4; pTM scores of 0.686) were predicted (Figure 5). Structural overlays revealed that the S protein of SF3 aligned with that of FD22 with the pruned-atom RMSD of 0.440 Å (952 atom pairs), and all-atom RMSD of 15.879 Å (1154 pairs); whereas SF184 had a lower pruned-atom RMSD of 0.361 Å (957 pairs) and all-atom RMSD of 7.172 Å (1157 pairs), indicating that the core folds of SF184 are closer to PDCoV FD22 than SF3.

### 3.5. SpDCoV Strains Were Not Isolated in ECEs or Cell Lines

We attempted to isolate SpDCoVs since the genetically related PDCoV propagated well in ECEs and several cell lines (LLC-PK1, ST, and DF-1) [19,22,31,32]. No CPE was observed in the four cell lines by 4 dpi. The P1 passage in Vero E6 yielded a weak positive result (Ct = 36.87), whereas all subsequent passages tested negative. However, no virus was isolated in these cell lines after three passages, as tested by the PDCoV RT-qPCR.

We inoculated the allantoic sac of 5-day-old ECEs to isolate the SpDCoVs from nine samples (SF3, SF9, SF80, SF88, SF94, SF104, SF126, SF135, and SF184). During the first two passages, no ECEs were found dead, and the allantoic fluids harvested at 5 dpi were negative by the panDelta RT-PCR. At passage three (P3), only one ECE inoculated with SF80 died at 2 dpi. The allantoic fluid was weakly positive for deltaCoV by panDelta RT-PCR and strongly positive for gammaCoV by the panGamma RT-PCR, suggesting that the gammaCoV SF80G, rather than the deltaCoV SF80 in the original sparrow feces, might replicate in the ECEs.

For the following virus isolation using ECEs, the SF80 fecal suspension was pre-treated with hyperimmune serum against TCoV to neutralize the gammaCoV before inoculation. The ECEs were inoculated with SF80 via two routes, the allantoic sac and CAM (Supplementary Table S2). At 5–7 dpi, the allantoic fluids tested weakly positive by RT-qPCR, with high Ct values (36–38, corresponding to 1–10 RNA copies/reaction calculated from the standard curve). In all ECE experiments, the negative and positive controls performed as expected: all PDCoV-inoculated ECEs died at 2–3 dpi, and their allantoic fluids tested strongly positive by RT-qPCR (Ct values 22–24, corresponding to 5 log_10_ RNA copies/reaction). Continuous passaging of SF80 in ECEs resulted in no significant decrease in Ct values, indicating no increase in viral RNA titer. Even after concentrating PDCoV RNA–positive allantoic fluids over 30-fold or 100-fold by ultracentrifugation, no PDCoV RNA was detected. These findings suggest that SF80 likely did not complete a full replication cycle and that no infectious viral particles were produced in the ECEs.

## 4. Discussion

Coronaviruses are a group of viruses that often cross species barriers and cause severe diseases and epidemics/pandemics in humans and livestock, such as severe acute respiratory syndrome (SARS)-CoV-1 and SARS-CoV-2, and porcine epidemic diarrhea virus (PEDV) [33,34]. The first PDCoV outbreak was reported in Ohio, USA [4]. Because house sparrows often visit farms and SpDCoVs closely related to PDCoVs were detected on farms [28], we surveyed for PDCoVs from fecal/cloacal swab and oropharyngeal swab samples collected from house sparrows near swine farms across various locations in Ohio during the winter of 2020–2021. DeltaCoVs were detected more frequently than gammaCoVs in fecal samples, whereas none were detected in the oropharyngeal swabs. These findings suggest that both gammaCoVs and deltaCoVs primarily shed in the feces or droppings of house sparrows. All sampled sparrows appeared clinically normal. These results suggest that sparrows may act as a natural reservoir or carrier for gammaCoVs and deltaCoVs, like other wild birds [18]. Although SF80 shared 100% nucleotide identity with PDCoV in the partial RdRp region (362-nt), it cannot be amplified by nested RT-PCR using PDCoV-specific primers targeting other genomic regions. In general, 3–7-day-old ECEs were very sensitive to PDCoV and the virus replicated efficiently in them [19,31]. Therefore, the inability of SF80 to replicate in ECEs indicate that this SpDCoV is probably quite different from PDCoV in the rest of genomic regions or the virus load in the original sample was extremely low.

We further sequenced the near-complete genomes of two representative SpDCoV strains (SF3 and SF184) that are genetically closest to the four ISU strains detected on swine barns/farms in 2017 in Illinois and Minnesota [28]. These results indicate that such SpDCoVs are widespread in house sparrows in the Midwest of the U.S. The forward primer and probe sequences used in our RT-qPCR assay match 100% to the M gene of SF3 and SF184, and the reverse primer had 3-nt and 2-nt mismatches to SF3 and SF184 sequences, respectively. This assay detected SF3 and SF184. So, this assay cannot differentiate PDCoV and SpDCoV when used to test environmental samples. In the future, differentiation assay can be developed to distinguish PDCoVs from SpDCoVs.

The donor identity of the PDCoV S gene remains unknown. The facts that recombinant PDCoVs carrying the S protein of SpDCoV HKU17 or the RBD of ISU73347 lose efficient replication in pigs [35], and PDCoVs have evolved specific INDELs in the S1 subunit (Figure 4) indicate that PDCoVs did not arise directly from any currently known SpDCoVs. Recently, deltaCoVs WS38 and WS31 detected from pigeon feces and drinking water of pigeons in 2022–2023 from a live poultry market in Shandong province of China shared the highest sequence identity at genomic level with SpDCoV HKU17, except for the S protein [17]. Interestingly, the S proteins of WS38 and WS31 not only showed the highest identity (87.9–88.3%) to PDCoV S proteins but also contain all the PDCoV-specific INDELs in the S1 subunit. Therefore, pigeon deltaCoVs may be involved in PDCoV evolution. Additionally, the S proteins of avian deltaCoVs and PDCoVs contain a 119–125-amino-acid extension at the N-terminal domain (NTD) compared with the S proteins of the viruses detected in ALCs and CFBs from a wet market in Guangxi in 2006. Aside from this NTD region, the remainder of the S protein (residues 129–1160 of PDCoV HKU15 and residues 4–1035 of ALC/CFB PDCoV S proteins) is 99% (1028/1032) identical, suggesting minimal host adaptation of PDCoV in ALCs/CFBs. Because pig organs were used to feed ALCs/CFBs at wet markets and molecular clock analyses estimated that PDCoV emerged around the 1990s [1], it is predicted that PDCoV was transmitted from pigs to ALCs/CFBs. In these later hosts, PDCoV likely replicated inefficiently and failed to establish sustained infection, as supported by the low viral RNA levels detected and the inability to recover complete viral genomes from the samples, PDCoV has only been detected in those market-traded ALCs/CFBs and not in wild populations [2], and there have been no new reports of PDCoV in these species since then. Therefore, continued surveillance of diverse wild animal species may illustrate clearer ecology and evolution of deltaCoVs.

Limitations of the current study: To detect and identify deltaCoVs and gammaCoVs, we designed new primers targeting a 400–450-nt region of the RdRp gene, based on sequences from dozens of gammaCoVs and deltaCoVs, respectively. This amplicon size allowed us to obtain sufficient sequence data for preliminary virus identification; however, they may be less sensitive compared with RT-PCR assays that use primers targeting shorter regions. As alphaCoVs and betaCoVs have not been reported in avian species, they were not targeted in this study. Recently, Drs. Linda Saif and Anastasia Vlasova laboratories at The Ohio State University, supported by WHO CoViNet, developed updated pan-CoV primers for the detection of all four genera of CoVs from different types of samples. These assays have been validated by several CoViNet reference laboratories and can be utilized for future surveillance studies.

## 5. Conclusions

Our results suggest that PDCoV-related SpDCoVs are widespread in the U.S. Midwest and that sparrows may play a significant role in the evolution and transmission of deltacoronaviruses. PDCoV infection in pigs can range from asymptomatic cases, commonly seen in adults, to severe diarrhea, particularly in neonatal pigs. Given the large global pig population and the frequent interactions between pigs and house sparrows—which can carry and transmit viruses to other avian species—the potential for PDCoV transmission between avian and mammalian hosts raises significant public health concerns. Continuous monitoring of this emerging zoonotic PDCoV in diverse wild animals, livestock, and humans is essential to mitigate the risk of future coronavirus pandemics.

## Figures and Tables

**Table 1:** Sparrow sample information and panDelta or panGamma RT-PCR results.

Farm No.	Collection Date (Yr.Mon.Date)	Sample No.	Positive DeltaCoV Samples ^2^	Positive DeltaCoV Rate (%)	Positive GammaCoV Samples ^2^	Positive GammaCoV Rate (%)
1	2020.12.09	SF1–12	SF3, 5, 7, 9, 10	5/12 (42)	SF11, 12	2/12 (17)
	2021.02.23	SF93–103	SF94, 97, 100	3/11 (27)		0/11 (0)
	2021.03.05	SF165–176		0/12 (0)		0/12 (0)
2	2020.12.17	SF13–23	SF13, 15, **17**, 18, 20	5/11 (45)	SF**17**	1/11 (9)
	2021.02.25	SF115–125		0/11 (0)	SF118	1/11 (9)
	2021.03.08	SF177–187	SF184	1/11 (9)		0/11 (0)
3	2020.12.18	SF24–34	SF24, 26, 28, 30, 32, 33	6/11 (55)		0/11 (0)
	2021.02.08	SF71–75		0/5 (0)		0/5 (0)
	2021.02.24	SF104–114	SF104	1/11 (9)		0/11 (0)
4	2021.01.12	SF35–45	SF38, 40, 42, 43	4/11 (36)		0/11 (0)
	2021.02.12	SF81–92	SF82, 88, 89	3/12 (25)	SF85	1/12 (8)
	2021.03.03	SF143–153		0/11 (0)	SF148	1/11 (9)
5	2021.01.19	SF46		0/1 (0)		0/1 (0)
6	2021.01.27	SF47–57	SF**54**, 55	2/11 (18)	SF51, **54**	2/11 (18)
	2021.02.11	SF76–80	SF**80**	1/5 (20)	SF**80**	1/5 (20)
	2021.03.02	SF137–142		0/6 (0)		0/6 (0)
7	2021.02.03	SF60–70	SF**60**, 64	2/11 (18)	SF**60**, 63	2/11 (18)
	2021.02.26	SF126–136	SF126, 127, 135	3/11 (27)		0/11 (0)
	2021.03.16	SF188–200		0/13 (0)		0/13 (0)
8	2021.03.04	SF154–164		0/11 (0)		0/11 (0)
NA ^1^	2021.02.02	SF58–59		0/2 (0)		0/2 (0)

^1^ In the Clintonville neighborhood of Columbus, OH, USA. ^2^ Sequenced representative samples are underlined, and samples were positive for both deltaCoV and gammaCoV are bolded.

## Data Availability

The data presented in this study are available in this article and on request from the corresponding author.

## Supplementary Materials

The following supporting information can be downloaded at: https://www.mdpi.com/article/10.3390/v17101326/s1. Figure S1A–H: ML phylogenetic trees (with 1000 bootstrap) of different genes; Table S1: Sequence diversity based on complete genomes, ORF1ab, S, E, M, N, NS6, NS7a, NS7b, and NS7c; Table S2: The inoculation of embryonated chicken eggs (ECEs) with SF80 for virus isolation; Table S3: The nucleotide sequence alignments of the S1 region of SpDCoVs, PDCoVs and closely related avian DCoVs; Table S4: Docking ranking metrics for SpDCoV/SF3 and SpDCoV/SF184 S proteins in complex with porcine APN as computed by HADDOCK2.4; Table S5A: The predicted interface interactions between SpDCoV SF3 and porcine APN; Table S5B: The predicted interface interactions between SpDCoV SF184 and porcine APN.

## Abbreviations

The following abbreviations are used in this manuscript:

Porcine peptidase N	pAPN
CoV	Coronavirus
CAM	Chorioallantoic membrane
ECE	Embryonated chicken egg
PDCoV	Porcine deltacoronavirus
SpDCoV	Sparrow deltacoronavirus
