# Supplementary material for: Porcine Deltacoronavirus-Related Viruses in House Sparrows
Source: Viruses. 2025 Sep 30;17(10):1326. doi: 10.3390/v17101326 (PMC12568082; doi:10.3390/v17101326)
Supplement: Supplementary file 1 [file viruses-17-01326-s001.zip › Figure S1.pptx]

## Slide 1
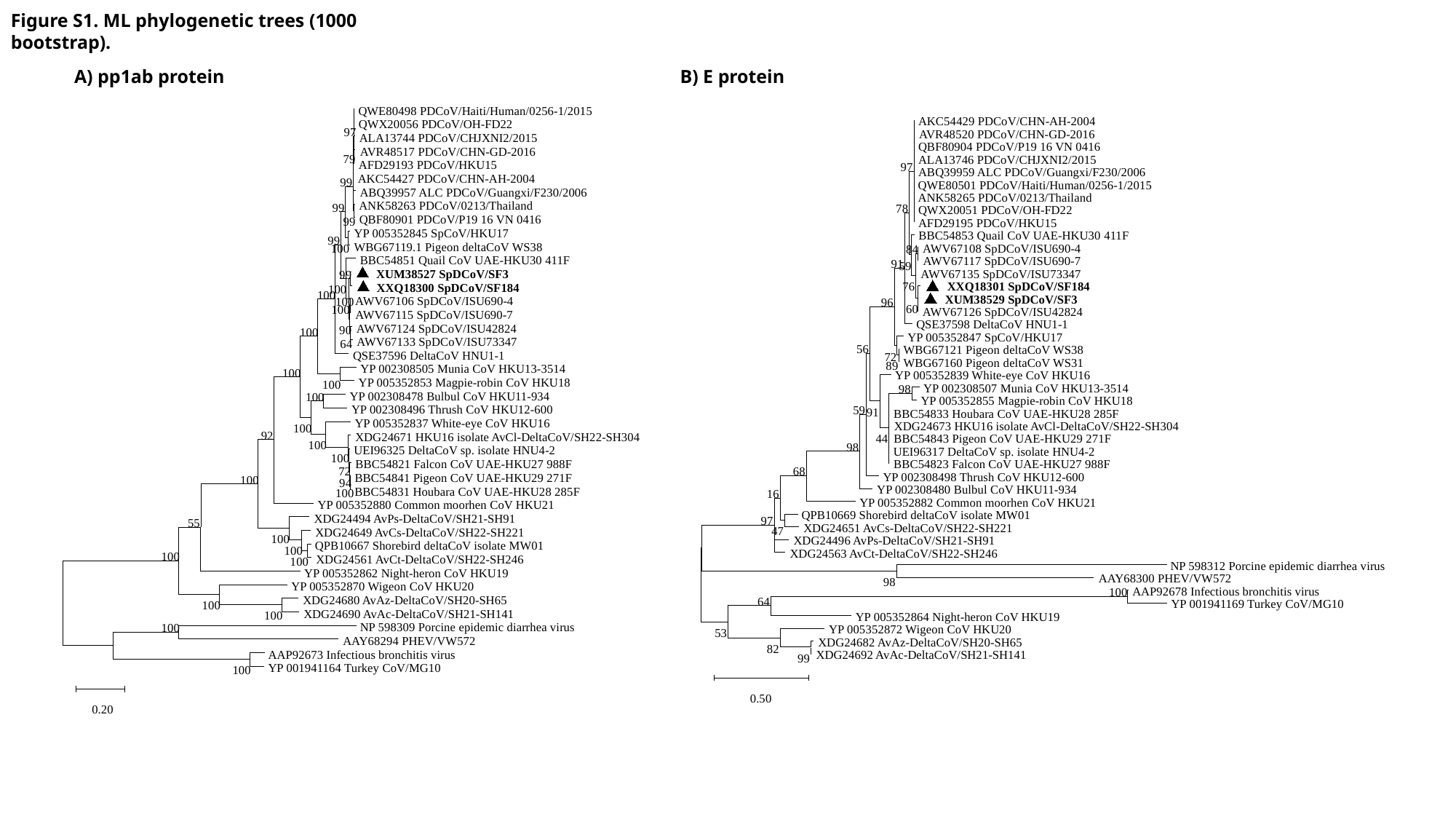

Figure S1. ML phylogenetic trees (1000 bootstrap).
A) pp1ab protein
B) E protein
 QWE80498 PDCoV/Haiti/Human/0256-1/2015
 QWX20056 PDCoV/OH-FD22
97
 ALA13744 PDCoV/CHJXNI2/2015
 AVR48517 PDCoV/CHN-GD-2016
79
 AFD29193 PDCoV/HKU15
 AKC54427 PDCoV/CHN-AH-2004
99
 ABQ39957 ALC PDCoV/Guangxi/F230/2006
 ANK58263 PDCoV/0213/Thailand
99
 QBF80901 PDCoV/P19 16 VN 0416
99
 YP 005352845 SpCoV/HKU17
99
 WBG67119.1 Pigeon deltaCoV WS38
100
 BBC54851 Quail CoV UAE-HKU30 411F
 XUM38527 SpDCoV/SF3
99
 XXQ18300 SpDCoV/SF184
100
100
 AWV67106 SpDCoV/ISU690-4
100
100
 AWV67115 SpDCoV/ISU690-7
 AWV67124 SpDCoV/ISU42824
90
100
 AWV67133 SpDCoV/ISU73347
64
 QSE37596 DeltaCoV HNU1-1
 YP 002308505 Munia CoV HKU13-3514
100
 YP 005352853 Magpie-robin CoV HKU18
100
 YP 002308478 Bulbul CoV HKU11-934
100
 YP 002308496 Thrush CoV HKU12-600
 YP 005352837 White-eye CoV HKU16
100
92
 XDG24671 HKU16 isolate AvCl-DeltaCoV/SH22-SH304
100
 UEI96325 DeltaCoV sp. isolate HNU4-2
100
 BBC54821 Falcon CoV UAE-HKU27 988F
72
 BBC54841 Pigeon CoV UAE-HKU29 271F
100
94
 BBC54831 Houbara CoV UAE-HKU28 285F
100
 YP 005352880 Common moorhen CoV HKU21
 XDG24494 AvPs-DeltaCoV/SH21-SH91
55
 XDG24649 AvCs-DeltaCoV/SH22-SH221
100
 QPB10667 Shorebird deltaCoV isolate MW01
100
100
 XDG24561 AvCt-DeltaCoV/SH22-SH246
100
 YP 005352862 Night-heron CoV HKU19
 YP 005352870 Wigeon CoV HKU20
 XDG24680 AvAz-DeltaCoV/SH20-SH65
100
 XDG24690 AvAc-DeltaCoV/SH21-SH141
100
 NP 598309 Porcine epidemic diarrhea virus
100
 AAY68294 PHEV/VW572
 AAP92673 Infectious bronchitis virus
 YP 001941164 Turkey CoV/MG10
100
0.20
 AKC54429 PDCoV/CHN-AH-2004
 AVR48520 PDCoV/CHN-GD-2016
 QBF80904 PDCoV/P19 16 VN 0416
 ALA13746 PDCoV/CHJXNI2/2015
97
 ABQ39959 ALC PDCoV/Guangxi/F230/2006
 QWE80501 PDCoV/Haiti/Human/0256-1/2015
 ANK58265 PDCoV/0213/Thailand
78
 QWX20051 PDCoV/OH-FD22
 AFD29195 PDCoV/HKU15
 BBC54853 Quail CoV UAE-HKU30 411F
 AWV67108 SpDCoV/ISU690-4
84
 AWV67117 SpDCoV/ISU690-7
91
59
 AWV67135 SpDCoV/ISU73347
76
 XXQ18301 SpDCoV/SF184
 XUM38529 SpDCoV/SF3
96
60
 AWV67126 SpDCoV/ISU42824
 QSE37598 DeltaCoV HNU1-1
 YP 005352847 SpCoV/HKU17
56
 WBG67121 Pigeon deltaCoV WS38
72
 WBG67160 Pigeon deltaCoV WS31
89
 YP 005352839 White-eye CoV HKU16
 YP 002308507 Munia CoV HKU13-3514
98
 YP 005352855 Magpie-robin CoV HKU18
59
91
 BBC54833 Houbara CoV UAE-HKU28 285F
 XDG24673 HKU16 isolate AvCl-DeltaCoV/SH22-SH304
44
 BBC54843 Pigeon CoV UAE-HKU29 271F
98
 UEI96317 DeltaCoV sp. isolate HNU4-2
 BBC54823 Falcon CoV UAE-HKU27 988F
68
 YP 002308498 Thrush CoV HKU12-600
 YP 002308480 Bulbul CoV HKU11-934
16
 YP 005352882 Common moorhen CoV HKU21
 QPB10669 Shorebird deltaCoV isolate MW01
97
 XDG24651 AvCs-DeltaCoV/SH22-SH221
47
 XDG24496 AvPs-DeltaCoV/SH21-SH91
 XDG24563 AvCt-DeltaCoV/SH22-SH246
 NP 598312 Porcine epidemic diarrhea virus
 AAY68300 PHEV/VW572
98
 AAP92678 Infectious bronchitis virus
100
64
 YP 001941169 Turkey CoV/MG10
 YP 005352864 Night-heron CoV HKU19
 YP 005352872 Wigeon CoV HKU20
53
 XDG24682 AvAz-DeltaCoV/SH20-SH65
82
 XDG24692 AvAc-DeltaCoV/SH21-SH141
99
0.50

## Slide 2
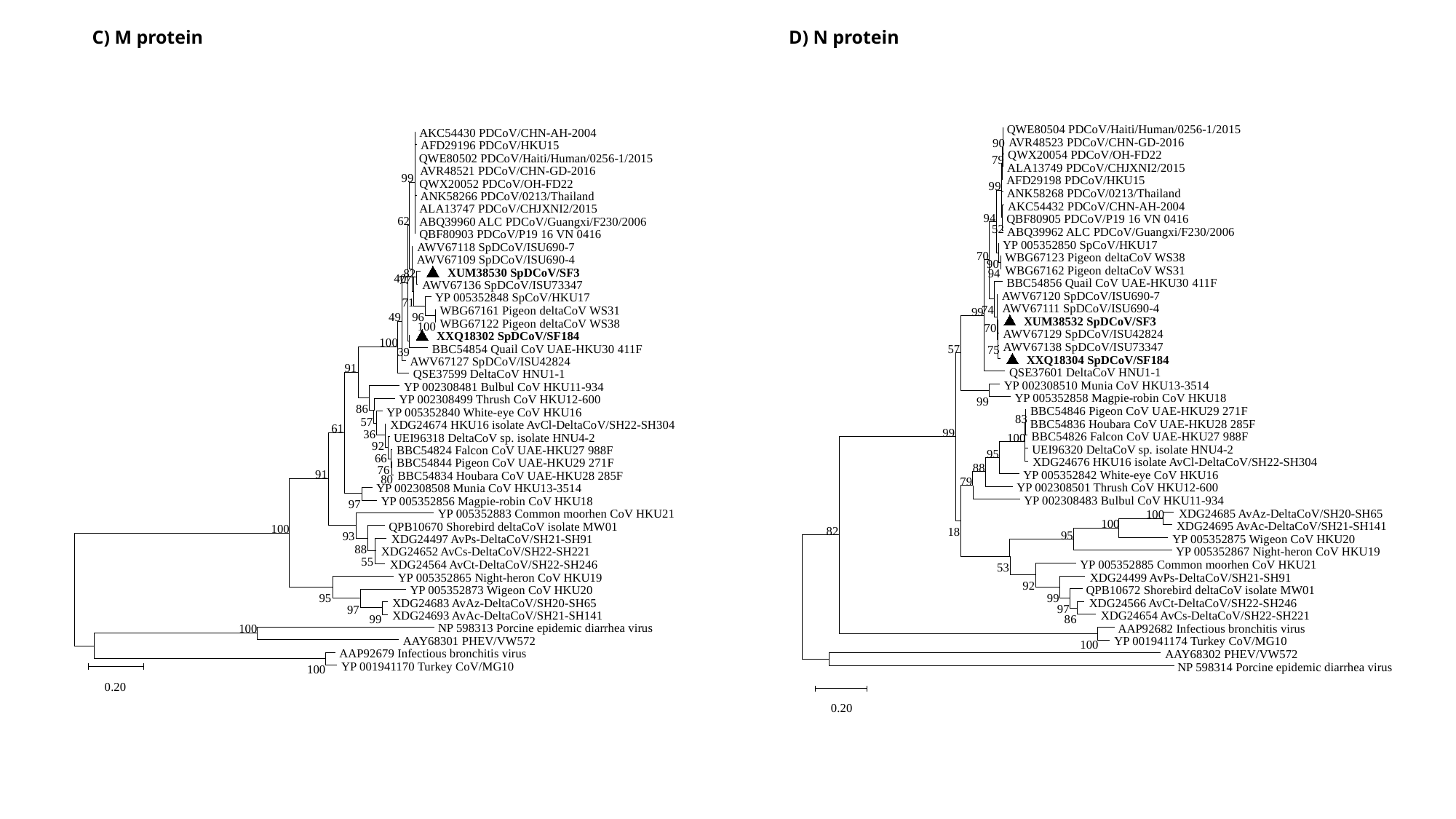

D) N protein
C) M protein
 QWE80504 PDCoV/Haiti/Human/0256-1/2015
 AVR48523 PDCoV/CHN-GD-2016
90
 QWX20054 PDCoV/OH-FD22
79
 ALA13749 PDCoV/CHJXNI2/2015
 AFD29198 PDCoV/HKU15
99
 ANK58268 PDCoV/0213/Thailand
 AKC54432 PDCoV/CHN-AH-2004
94
 QBF80905 PDCoV/P19 16 VN 0416
52
 ABQ39962 ALC PDCoV/Guangxi/F230/2006
 YP 005352850 SpCoV/HKU17
70
 WBG67123 Pigeon deltaCoV WS38
90
 WBG67162 Pigeon deltaCoV WS31
94
 BBC54856 Quail CoV UAE-HKU30 411F
 AWV67120 SpDCoV/ISU690-7
 AWV67111 SpDCoV/ISU690-4
74
99
 XUM38532 SpDCoV/SF3
70
 AWV67129 SpDCoV/ISU42824
 AWV67138 SpDCoV/ISU73347
57
75
 XXQ18304 SpDCoV/SF184
 QSE37601 DeltaCoV HNU1-1
 YP 002308510 Munia CoV HKU13-3514
 YP 005352858 Magpie-robin CoV HKU18
99
 BBC54846 Pigeon CoV UAE-HKU29 271F
83
 BBC54836 Houbara CoV UAE-HKU28 285F
99
 BBC54826 Falcon CoV UAE-HKU27 988F
100
 UEI96320 DeltaCoV sp. isolate HNU4-2
95
 XDG24676 HKU16 isolate AvCl-DeltaCoV/SH22-SH304
88
 YP 005352842 White-eye CoV HKU16
79
 YP 002308501 Thrush CoV HKU12-600
 YP 002308483 Bulbul CoV HKU11-934
 XDG24685 AvAz-DeltaCoV/SH20-SH65
100
100
 XDG24695 AvAc-DeltaCoV/SH21-SH141
82
18
95
 YP 005352875 Wigeon CoV HKU20
 YP 005352867 Night-heron CoV HKU19
 YP 005352885 Common moorhen CoV HKU21
53
 XDG24499 AvPs-DeltaCoV/SH21-SH91
92
 QPB10672 Shorebird deltaCoV isolate MW01
99
 XDG24566 AvCt-DeltaCoV/SH22-SH246
97
 XDG24654 AvCs-DeltaCoV/SH22-SH221
86
 AAP92682 Infectious bronchitis virus
 YP 001941174 Turkey CoV/MG10
100
 AAY68302 PHEV/VW572
 NP 598314 Porcine epidemic diarrhea virus
0.20
 AKC54430 PDCoV/CHN-AH-2004
 AFD29196 PDCoV/HKU15
 QWE80502 PDCoV/Haiti/Human/0256-1/2015
 AVR48521 PDCoV/CHN-GD-2016
99
 QWX20052 PDCoV/OH-FD22
 ANK58266 PDCoV/0213/Thailand
 ALA13747 PDCoV/CHJXNI2/2015
62
 ABQ39960 ALC PDCoV/Guangxi/F230/2006
 QBF80903 PDCoV/P19 16 VN 0416
 AWV67118 SpDCoV/ISU690-7
 AWV67109 SpDCoV/ISU690-4
 XUM38530 SpDCoV/SF3
82
40
77
 AWV67136 SpDCoV/ISU73347
 YP 005352848 SpCoV/HKU17
71
 WBG67161 Pigeon deltaCoV WS31
49
96
 WBG67122 Pigeon deltaCoV WS38
100
 XXQ18302 SpDCoV/SF184
100
 BBC54854 Quail CoV UAE-HKU30 411F
39
 AWV67127 SpDCoV/ISU42824
91
 QSE37599 DeltaCoV HNU1-1
 YP 002308481 Bulbul CoV HKU11-934
 YP 002308499 Thrush CoV HKU12-600
86
 YP 005352840 White-eye CoV HKU16
57
 XDG24674 HKU16 isolate AvCl-DeltaCoV/SH22-SH304
61
36
 UEI96318 DeltaCoV sp. isolate HNU4-2
92
 BBC54824 Falcon CoV UAE-HKU27 988F
66
 BBC54844 Pigeon CoV UAE-HKU29 271F
76
91
 BBC54834 Houbara CoV UAE-HKU28 285F
80
 YP 002308508 Munia CoV HKU13-3514
 YP 005352856 Magpie-robin CoV HKU18
97
 YP 005352883 Common moorhen CoV HKU21
 QPB10670 Shorebird deltaCoV isolate MW01
100
93
 XDG24497 AvPs-DeltaCoV/SH21-SH91
88
 XDG24652 AvCs-DeltaCoV/SH22-SH221
55
 XDG24564 AvCt-DeltaCoV/SH22-SH246
 YP 005352865 Night-heron CoV HKU19
 YP 005352873 Wigeon CoV HKU20
95
 XDG24683 AvAz-DeltaCoV/SH20-SH65
97
 XDG24693 AvAc-DeltaCoV/SH21-SH141
99
 NP 598313 Porcine epidemic diarrhea virus
100
 AAY68301 PHEV/VW572
 AAP92679 Infectious bronchitis virus
 YP 001941170 Turkey CoV/MG10
100
0.20
0.20

## Slide 3
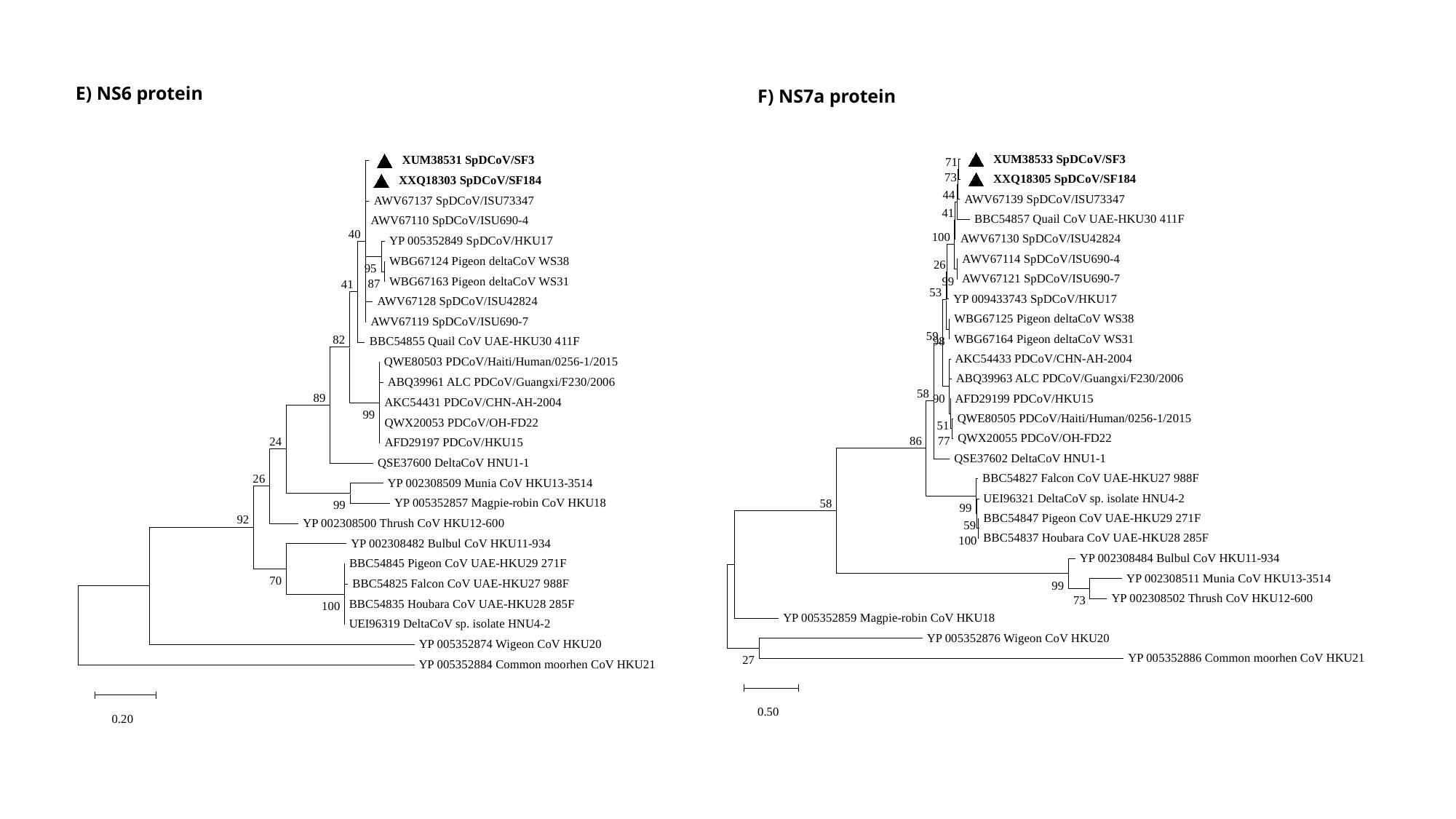

E) NS6 protein
F) NS7a protein
 XUM38533 SpDCoV/SF3
71
73
 XXQ18305 SpDCoV/SF184
44
 AWV67139 SpDCoV/ISU73347
41
 BBC54857 Quail CoV UAE-HKU30 411F
100
 AWV67130 SpDCoV/ISU42824
 AWV67114 SpDCoV/ISU690-4
26
 AWV67121 SpDCoV/ISU690-7
99
53
 YP 009433743 SpDCoV/HKU17
 WBG67125 Pigeon deltaCoV WS38
59
 WBG67164 Pigeon deltaCoV WS31
98
 AKC54433 PDCoV/CHN-AH-2004
 ABQ39963 ALC PDCoV/Guangxi/F230/2006
58
90
 AFD29199 PDCoV/HKU15
 QWE80505 PDCoV/Haiti/Human/0256-1/2015
51
 QWX20055 PDCoV/OH-FD22
86
77
 QSE37602 DeltaCoV HNU1-1
 BBC54827 Falcon CoV UAE-HKU27 988F
 UEI96321 DeltaCoV sp. isolate HNU4-2
58
99
 BBC54847 Pigeon CoV UAE-HKU29 271F
59
 BBC54837 Houbara CoV UAE-HKU28 285F
100
 YP 002308484 Bulbul CoV HKU11-934
 YP 002308511 Munia CoV HKU13-3514
99
 YP 002308502 Thrush CoV HKU12-600
73
 YP 005352859 Magpie-robin CoV HKU18
 YP 005352876 Wigeon CoV HKU20
 YP 005352886 Common moorhen CoV HKU21
27
0.50
 XUM38531 SpDCoV/SF3
 XXQ18303 SpDCoV/SF184
 AWV67137 SpDCoV/ISU73347
 AWV67110 SpDCoV/ISU690-4
40
 YP 005352849 SpDCoV/HKU17
 WBG67124 Pigeon deltaCoV WS38
95
 WBG67163 Pigeon deltaCoV WS31
87
41
 AWV67128 SpDCoV/ISU42824
 AWV67119 SpDCoV/ISU690-7
82
 BBC54855 Quail CoV UAE-HKU30 411F
 QWE80503 PDCoV/Haiti/Human/0256-1/2015
 ABQ39961 ALC PDCoV/Guangxi/F230/2006
89
 AKC54431 PDCoV/CHN-AH-2004
99
 QWX20053 PDCoV/OH-FD22
24
 AFD29197 PDCoV/HKU15
 QSE37600 DeltaCoV HNU1-1
26
 YP 002308509 Munia CoV HKU13-3514
 YP 005352857 Magpie-robin CoV HKU18
99
92
 YP 002308500 Thrush CoV HKU12-600
 YP 002308482 Bulbul CoV HKU11-934
 BBC54845 Pigeon CoV UAE-HKU29 271F
70
 BBC54825 Falcon CoV UAE-HKU27 988F
 BBC54835 Houbara CoV UAE-HKU28 285F
100
 UEI96319 DeltaCoV sp. isolate HNU4-2
 YP 005352874 Wigeon CoV HKU20
 YP 005352884 Common moorhen CoV HKU21
0.20

## Slide 4
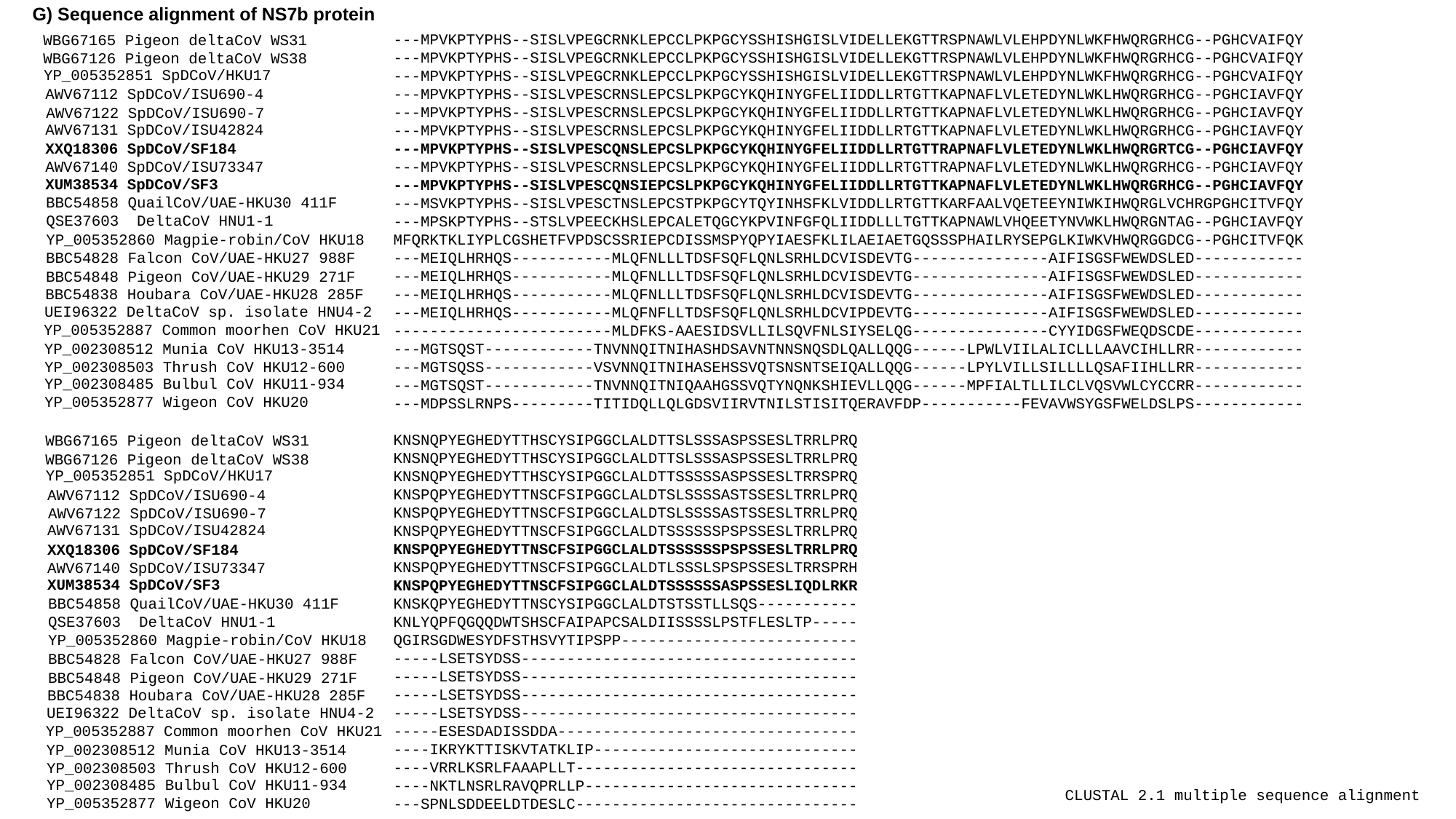

G) Sequence alignment of NS7b protein
---MPVKPTYPHS--SISLVPEGCRNKLEPCCLPKPGCYSSHISHGISLVIDELLEKGTTRSPNAWLVLEHPDYNLWKFHWQRGRHCG--PGHCVAIFQY
---MPVKPTYPHS--SISLVPEGCRNKLEPCCLPKPGCYSSHISHGISLVIDELLEKGTTRSPNAWLVLEHPDYNLWKFHWQRGRHCG--PGHCVAIFQY
---MPVKPTYPHS--SISLVPEGCRNKLEPCCLPKPGCYSSHISHGISLVIDELLEKGTTRSPNAWLVLEHPDYNLWKFHWQRGRHCG--PGHCVAIFQY
---MPVKPTYPHS--SISLVPESCRNSLEPCSLPKPGCYKQHINYGFELIIDDLLRTGTTKAPNAFLVLETEDYNLWKLHWQRGRHCG--PGHCIAVFQY
---MPVKPTYPHS--SISLVPESCRNSLEPCSLPKPGCYKQHINYGFELIIDDLLRTGTTKAPNAFLVLETEDYNLWKLHWQRGRHCG--PGHCIAVFQY
---MPVKPTYPHS--SISLVPESCRNSLEPCSLPKPGCYKQHINYGFELIIDDLLRTGTTKAPNAFLVLETEDYNLWKLHWQRGRHCG--PGHCIAVFQY
---MPVKPTYPHS--SISLVPESCQNSLEPCSLPKPGCYKQHINYGFELIIDDLLRTGTTRAPNAFLVLETEDYNLWKLHWQRGRTCG--PGHCIAVFQY
---MPVKPTYPHS--SISLVPESCRNSLEPCSLPKPGCYKQHINYGFELIIDDLLRTGTTRAPNAFLVLETEDYNLWKLHWQRGRHCG--PGHCIAVFQY
---MPVKPTYPHS--SISLVPESCQNSIEPCSLPKPGCYKQHINYGFELIIDDLLRTGTTKAPNAFLVLETEDYNLWKLHWQRGRHCG--PGHCIAVFQY
---MSVKPTYPHS--SISLVPESCTNSLEPCSTPKPGCYTQYINHSFKLVIDDLLRTGTTKARFAALVQETEEYNIWKIHWQRGLVCHRGPGHCITVFQY
---MPSKPTYPHS--STSLVPEECKHSLEPCALETQGCYKPVINFGFQLIIDDLLLTGTTKAPNAWLVHQEETYNVWKLHWQRGNTAG--PGHCIAVFQY
MFQRKTKLIYPLCGSHETFVPDSCSSRIEPCDISSMSPYQPYIAESFKLILAEIAETGQSSSPHAILRYSEPGLKIWKVHWQRGGDCG--PGHCITVFQK
---MEIQLHRHQS-----------MLQFNLLLTDSFSQFLQNLSRHLDCVISDEVTG---------------AIFISGSFWEWDSLED------------
---MEIQLHRHQS-----------MLQFNLLLTDSFSQFLQNLSRHLDCVISDEVTG---------------AIFISGSFWEWDSLED------------
---MEIQLHRHQS-----------MLQFNLLLTDSFSQFLQNLSRHLDCVISDEVTG---------------AIFISGSFWEWDSLED------------
---MEIQLHRHQS-----------MLQFNFLLTDSFSQFLQNLSRHLDCVIPDEVTG---------------AIFISGSFWEWDSLED------------
------------------------MLDFKS-AAESIDSVLLILSQVFNLSIYSELQG---------------CYYIDGSFWEQDSCDE------------
---MGTSQST------------TNVNNQITNIHASHDSAVNTNNSNQSDLQALLQQG------LPWLVIILALICLLLAAVCIHLLRR------------
---MGTSQSS------------VSVNNQITNIHASEHSSVQTSNSNTSEIQALLQQG------LPYLVILLSILLLLQSAFIIHLLRR------------
---MGTSQST------------TNVNNQITNIQAAHGSSVQTYNQNKSHIEVLLQQG------MPFIALTLLILCLVQSVWLCYCCRR------------
---MDPSSLRNPS---------TITIDQLLQLGDSVIIRVTNILSTISITQERAVFDP-----------FEVAVWSYGSFWELDSLPS------------
KNSNQPYEGHEDYTTHSCYSIPGGCLALDTTSLSSSASPSSESLTRRLPRQ
KNSNQPYEGHEDYTTHSCYSIPGGCLALDTTSLSSSASPSSESLTRRLPRQ
KNSNQPYEGHEDYTTHSCYSIPGGCLALDTTSSSSSASPSSESLTRRSPRQ
KNSPQPYEGHEDYTTNSCFSIPGGCLALDTSLSSSSASTSSESLTRRLPRQ
KNSPQPYEGHEDYTTNSCFSIPGGCLALDTSLSSSSASTSSESLTRRLPRQ
KNSPQPYEGHEDYTTNSCFSIPGGCLALDTSSSSSSPSPSSESLTRRLPRQ
KNSPQPYEGHEDYTTNSCFSIPGGCLALDTSSSSSSPSPSSESLTRRLPRQ
KNSPQPYEGHEDYTTNSCFSIPGGCLALDTLSSSLSPSPSSESLTRRSPRH
KNSPQPYEGHEDYTTNSCFSIPGGCLALDTSSSSSSASPSSESLIQDLRKR
KNSKQPYEGHEDYTTNSCYSIPGGCLALDTSTSSTLLSQS-----------
KNLYQPFQGQQDWTSHSCFAIPAPCSALDIISSSSLPSTFLESLTP-----
QGIRSGDWESYDFSTHSVYTIPSPP--------------------------
-----LSETSYDSS-------------------------------------
-----LSETSYDSS-------------------------------------
-----LSETSYDSS-------------------------------------
-----LSETSYDSS-------------------------------------
-----ESESDADISSDDA---------------------------------
----IKRYKTTISKVTATKLIP-----------------------------
----VRRLKSRLFAAAPLLT-------------------------------
----NKTLNSRLRAVQPRLLP------------------------------
---SPNLSDDEELDTDESLC-------------------------------
WBG67165 Pigeon deltaCoV WS31
WBG67126 Pigeon deltaCoV WS38
 YP_005352851 SpDCoV/HKU17
AWV67112 SpDCoV/ISU690-4
 AWV67122 SpDCoV/ISU690-7
AWV67131 SpDCoV/ISU42824
XXQ18306 SpDCoV/SF184
AWV67140 SpDCoV/ISU73347
XUM38534 SpDCoV/SF3
 BBC54858 QuailCoV/UAE-HKU30 411F
 QSE37603 DeltaCoV HNU1-1
 YP_005352860 Magpie-robin/CoV HKU18
 BBC54828 Falcon CoV/UAE-HKU27 988F
 BBC54848 Pigeon CoV/UAE-HKU29 271F
 BBC54838 Houbara CoV/UAE-HKU28 285F
 UEI96322 DeltaCoV sp. isolate HNU4-2
 YP_005352887 Common moorhen CoV HKU21
 YP_002308512 Munia CoV HKU13-3514
 YP_002308503 Thrush CoV HKU12-600
 YP_002308485 Bulbul CoV HKU11-934
 YP_005352877 Wigeon CoV HKU20
WBG67165 Pigeon deltaCoV WS31
WBG67126 Pigeon deltaCoV WS38
 YP_005352851 SpDCoV/HKU17
AWV67112 SpDCoV/ISU690-4
 AWV67122 SpDCoV/ISU690-7
AWV67131 SpDCoV/ISU42824
XXQ18306 SpDCoV/SF184
AWV67140 SpDCoV/ISU73347
XUM38534 SpDCoV/SF3
 BBC54858 QuailCoV/UAE-HKU30 411F
 QSE37603 DeltaCoV HNU1-1
 YP_005352860 Magpie-robin/CoV HKU18
 BBC54828 Falcon CoV/UAE-HKU27 988F
 BBC54848 Pigeon CoV/UAE-HKU29 271F
 BBC54838 Houbara CoV/UAE-HKU28 285F
 UEI96322 DeltaCoV sp. isolate HNU4-2
 YP_005352887 Common moorhen CoV HKU21
 YP_002308512 Munia CoV HKU13-3514
 YP_002308503 Thrush CoV HKU12-600
 YP_002308485 Bulbul CoV HKU11-934
 YP_005352877 Wigeon CoV HKU20
CLUSTAL 2.1 multiple sequence alignment

## Slide 5
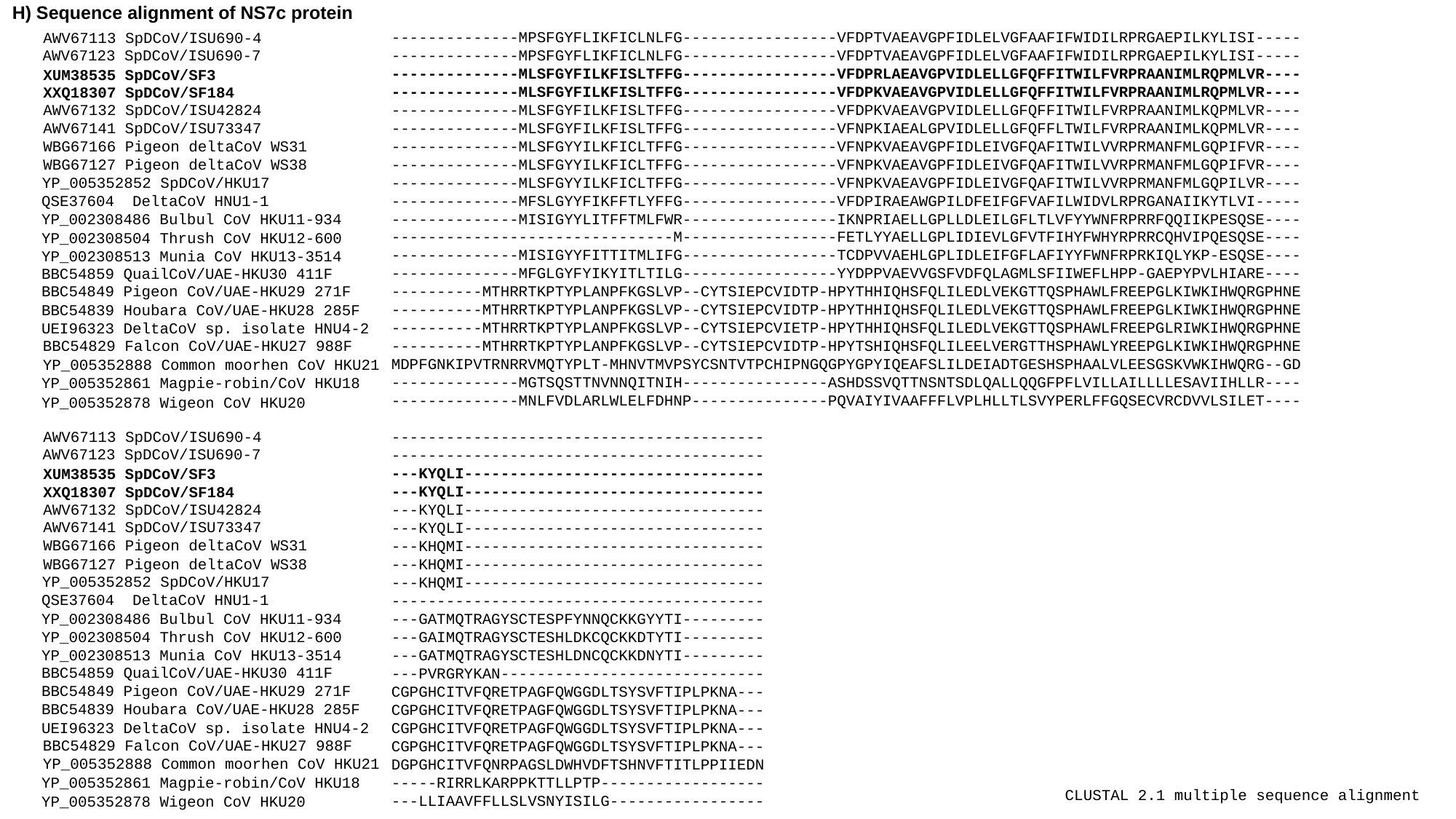

H) Sequence alignment of NS7c protein
--------------MPSFGYFLIKFICLNLFG-----------------VFDPTVAEAVGPFIDLELVGFAAFIFWIDILRPRGAEPILKYLISI-----
--------------MPSFGYFLIKFICLNLFG-----------------VFDPTVAEAVGPFIDLELVGFAAFIFWIDILRPRGAEPILKYLISI-----
--------------MLSFGYFILKFISLTFFG-----------------VFDPRLAEAVGPVIDLELLGFQFFITWILFVRPRAANIMLRQPMLVR----
--------------MLSFGYFILKFISLTFFG-----------------VFDPKVAEAVGPVIDLELLGFQFFITWILFVRPRAANIMLRQPMLVR----
--------------MLSFGYFILKFISLTFFG-----------------VFDPKVAEAVGPVIDLELLGFQFFITWILFVRPRAANIMLKQPMLVR----
--------------MLSFGYFILKFISLTFFG-----------------VFNPKIAEALGPVIDLELLGFQFFLTWILFVRPRAANIMLKQPMLVR----
--------------MLSFGYYILKFICLTFFG-----------------VFNPKVAEAVGPFIDLEIVGFQAFITWILVVRPRMANFMLGQPIFVR----
--------------MLSFGYYILKFICLTFFG-----------------VFNPKVAEAVGPFIDLEIVGFQAFITWILVVRPRMANFMLGQPIFVR----
--------------MLSFGYYILKFICLTFFG-----------------VFNPKVAEAVGPFIDLEIVGFQAFITWILVVRPRMANFMLGQPILVR----
--------------MFSLGYYFIKFFTLYFFG-----------------VFDPIRAEAWGPILDFEIFGFVAFILWIDVLRPRGANAIIKYTLVI-----
--------------MISIGYYLITFFTMLFWR-----------------IKNPRIAELLGPLLDLEILGFLTLVFYYWNFRPRRFQQIIKPESQSE----
-------------------------------M-----------------FETLYYAELLGPLIDIEVLGFVTFIHYFWHYRPRRCQHVIPQESQSE----
--------------MISIGYYFITTITMLIFG-----------------TCDPVVAEHLGPLIDLEIFGFLAFIYYFWNFRPRKIQLYKP-ESQSE----
--------------MFGLGYFYIKYITLTILG-----------------YYDPPVAEVVGSFVDFQLAGMLSFIIWEFLHPP-GAEPYPVLHIARE----
----------MTHRRTKPTYPLANPFKGSLVP--CYTSIEPCVIDTP-HPYTHHIQHSFQLILEDLVEKGTTQSPHAWLFREEPGLKIWKIHWQRGPHNE
----------MTHRRTKPTYPLANPFKGSLVP--CYTSIEPCVIDTP-HPYTHHIQHSFQLILEDLVEKGTTQSPHAWLFREEPGLKIWKIHWQRGPHNE
----------MTHRRTKPTYPLANPFKGSLVP--CYTSIEPCVIETP-HPYTHHIQHSFQLILEDLVEKGTTQSPHAWLFREEPGLRIWKIHWQRGPHNE
----------MTHRRTKPTYPLANPFKGSLVP--CYTSIEPCVIDTP-HPYTSHIQHSFQLILEELVERGTTHSPHAWLYREEPGLKIWKIHWQRGPHNE
MDPFGNKIPVTRNRRVMQTYPLT-MHNVTMVPSYCSNTVTPCHIPNGQGPYGPYIQEAFSLILDEIADTGESHSPHAALVLEESGSKVWKIHWQRG--GD
--------------MGTSQSTTNVNNQITNIH----------------ASHDSSVQTTNSNTSDLQALLQQGFPFLVILLAILLLLESAVIIHLLR----
--------------MNLFVDLARLWLELFDHNP---------------PQVAIYIVAAFFFLVPLHLLTLSVYPERLFFGQSECVRCDVVLSILET----
-----------------------------------------
-----------------------------------------
---KYQLI---------------------------------
---KYQLI---------------------------------
---KYQLI---------------------------------
---KYQLI---------------------------------
---KHQMI---------------------------------
---KHQMI---------------------------------
---KHQMI---------------------------------
-----------------------------------------
---GATMQTRAGYSCTESPFYNNQCKKGYYTI---------
---GAIMQTRAGYSCTESHLDKCQCKKDTYTI---------
---GATMQTRAGYSCTESHLDNCQCKKDNYTI---------
---PVRGRYKAN-----------------------------
CGPGHCITVFQRETPAGFQWGGDLTSYSVFTIPLPKNA---
CGPGHCITVFQRETPAGFQWGGDLTSYSVFTIPLPKNA---
CGPGHCITVFQRETPAGFQWGGDLTSYSVFTIPLPKNA---
CGPGHCITVFQRETPAGFQWGGDLTSYSVFTIPLPKNA---
DGPGHCITVFQNRPAGSLDWHVDFTSHNVFTITLPPIIEDN
-----RIRRLKARPPKTTLLPTP------------------
---LLIAAVFFLLSLVSNYISILG-----------------
AWV67113 SpDCoV/ISU690-4
 AWV67123 SpDCoV/ISU690-7
XUM38535 SpDCoV/SF3
XXQ18307 SpDCoV/SF184
AWV67132 SpDCoV/ISU42824
AWV67141 SpDCoV/ISU73347
WBG67166 Pigeon deltaCoV WS31
WBG67127 Pigeon deltaCoV WS38
 YP_005352852 SpDCoV/HKU17
 QSE37604 DeltaCoV HNU1-1
 YP_002308486 Bulbul CoV HKU11-934
 YP_002308504 Thrush CoV HKU12-600
 YP_002308513 Munia CoV HKU13-3514
 BBC54859 QuailCoV/UAE-HKU30 411F
 BBC54849 Pigeon CoV/UAE-HKU29 271F
 BBC54839 Houbara CoV/UAE-HKU28 285F
 UEI96323 DeltaCoV sp. isolate HNU4-2
 BBC54829 Falcon CoV/UAE-HKU27 988F
 YP_005352888 Common moorhen CoV HKU21
 YP_005352861 Magpie-robin/CoV HKU18
 YP_005352878 Wigeon CoV HKU20
AWV67113 SpDCoV/ISU690-4
 AWV67123 SpDCoV/ISU690-7
XUM38535 SpDCoV/SF3
XXQ18307 SpDCoV/SF184
AWV67132 SpDCoV/ISU42824
AWV67141 SpDCoV/ISU73347
WBG67166 Pigeon deltaCoV WS31
WBG67127 Pigeon deltaCoV WS38
 YP_005352852 SpDCoV/HKU17
 QSE37604 DeltaCoV HNU1-1
 YP_002308486 Bulbul CoV HKU11-934
 YP_002308504 Thrush CoV HKU12-600
 YP_002308513 Munia CoV HKU13-3514
 BBC54859 QuailCoV/UAE-HKU30 411F
 BBC54849 Pigeon CoV/UAE-HKU29 271F
 BBC54839 Houbara CoV/UAE-HKU28 285F
 UEI96323 DeltaCoV sp. isolate HNU4-2
 BBC54829 Falcon CoV/UAE-HKU27 988F
 YP_005352888 Common moorhen CoV HKU21
 YP_005352861 Magpie-robin/CoV HKU18
 YP_005352878 Wigeon CoV HKU20
CLUSTAL 2.1 multiple sequence alignment
