# Supplementary material for: Porcine Deltacoronavirus-Related Viruses in House Sparrows
Source: Viruses. 2025 Sep 30;17(10):1326. doi: 10.3390/v17101326 (PMC12568082; doi:10.3390/v17101326)
Supplement: Supplementary file 1 [file viruses-17-01326-s001.zip › Supplementary Table 2.pdf]

Supplementary Table 2. The inoculation of embryonated chicken eggs (ECEs) with SF80 for virus isolation.

Passage No.	ECE age (day)	Inoculation route [1]	Number of ECEs [2]	Harvest time (dpi)	RT-qPCR results ECE no.: positive sample Ct	PDCoV as positive control [4]	PBS as negative control
P1	6	A and B	A: 6; B: 3	5	A[3]: Negative; B[3]: 36.00	A[2]: 24.9; B[2]: 25.2	A[2]: Negative; B[2]: Negative
P1	7	A	6	6	Negative	ND [5]	ND
P2	6	A and B	A: 5; B: 10	6	A[3]: Negative; B[3]: 35.86	A[2]: 36.83	A[2]: 37.2
P3	5	A and B	A: 4; B: 7	5	A[3]: Negative; B[3]: 37.95	ND	A: Negative
P3	5	A and B	A: 4; B: 6	6	A[3]: Negative; B[3]: 36.62	ND	ND
P4	7	B	5	6	B_1: 37.20; B_3: 37.32	ND	B: Negative
P4	7	B	4	7	Negative	ND	ND
P4	10	A and B	A: 4; B: 4	5	Negative	ND	A: Negative
P4	10	A and B	A: 4; B: 4	6	A_1: 36.75; A_4: 36.89	ND	ND
P5	4	B	8	5	Negative	ND	B: Negative
P5	5	B	8	6	B_2: 35.85; B_3: 36.04	ND	ND
P5	10	A and B	A: 4; B: 3	6	A_3: 36.73; B: Negative;	ND	A: Negative; B: Negative
P5	10	A and B	A: 4; B: 3	7	Negative	ND	ND

1. Inoculation routes: Allantoic sac (A) or chorioallantoic membrane (CAM) (B)
2. The number used for virus isolation minus dead ones within 24 hpi, probably due to technical errors.
3. Combined allantoic fluid samples from several SpDCoV-inoculated ECEs from the save group.
4. For P1 and P2, 1:10 and 1:1000 diluted PDCoV FD22 strain (7.8 log10 TCID50/mL) was the positive control.
5. ND: not done. Positive control PDCoV was not included from P3 to avoid potential contamination. Negative
