# Supplementary material for: Porcine Deltacoronavirus-Related Viruses in House Sparrows
Source: Viruses. 2025 Sep 30;17(10):1326. doi: 10.3390/v17101326 (PMC12568082; doi:10.3390/v17101326)
Supplement: Supplementary file 1 [file viruses-17-01326-s001.zip › Supplementary Table 4.pdf]

Supplementary Table 4. Docking ranking metrics for SpDCoV/SF3 and SpDCoV/SF184 S proteins in complex with porcine APN as computed by HADDOCK2.4.

Strain	Ranking	HADDOCK score	Cluster size	RMSD from the overall lowest-energy structure	Z-Score
SpDCoV/SF3	Top 2*	-93.2 ± 10.6	12	5.4 ± 1.2	-1.0
SpDCoV/SF184	Top 1	-141.5 ± 4.5	13	0.9 ± 0.5	-2.0

Footnote:

* For SpDCoV/SF3, the Top 2 cluster was selected based on its better convergence (RMSD = 5.4 ± 1.2 Å) compared with the Top 1 (RMSD =12.0 ± 1.2 Å, Cluster size=12). Although the Top 1 had a slightly more favorable HADDOCK score (−103.6 ± 4.1 vs. -93.2 ± 10.6), its poorer convergence indicated by its higher RMSD undermines its reliability.
